# Supplementary material for: Evaluation of plasma anti-GPL-core IgA and IgG for diagnosis of disseminated non-tuberculous mycobacteria infection
Source: PLoS One. 2020 Nov 30;15(11):e0242598. doi: 10.1371/journal.pone.0242598 (PMC7703992; doi:10.1371/journal.pone.0242598)
Supplement: S1 Fig — Human reference plasma was diluted at 10, 7.5, 5, 2.5, and 1.25 AU/ml. Optical density (O.D.) of each concentration was plotted before analyzing with linear regression by using Prism GraphPad Software. (DOCX) [file pone.0242598.s001.docx]

**S1 Fig. Human plasma anti-GPL-core IgG standard curve analyzed by linear regression.** Human reference plasma was diluted at 10, 7.5, 5, 2.5, and 1.25 AU/ml. Optical density (O.D.) of each concentration was plotted before analyzing with linear regression by using Prism GraphPad Software.
