# Supplementary material for: Evaluation of plasma anti-GPL-core IgA and IgG for diagnosis of disseminated non-tuberculous mycobacteria infection
Source: PLoS One. 2020 Nov 30;15(11):e0242598. doi: 10.1371/journal.pone.0242598 (PMC7703992; doi:10.1371/journal.pone.0242598)
Supplement: S2 Fig — Receiver operating characteristic (ROC) curve analysis was applied to determine area under curve (AUC) and positive cut-off for NTM infection diagnosis by detecting anti-GPL core IgA (A) or IgG level (B). Red arrow represents cut-off point on ROC curve at 90% sensitivity. Blue arrow represents cut-off point on ROC curve at 90% specificity. (DOCX) [file pone.0242598.s002.docx]

**S2 Fig. Determination of positive cut-off for NTM diagnosis by detecting level of plasma anti-GPL core IgA or IgG.** Receiver operating characteristic (ROC) curve analysis was applied to determine area under curve (AUC) and positive cut-off for NTM infection diagnosis by detecting anti-GPL core IgA **(A)** or IgG level **(B)**. Red arrow represents cut-off point on ROC curve at 90% sensitivity. Blue arrow represents cut-off point on ROC curve at 90% specificity.
