# Supplementary material for: Evaluation of plasma anti-GPL-core IgA and IgG for diagnosis of disseminated non-tuberculous mycobacteria infection
Source: PLoS One. 2020 Nov 30;15(11):e0242598. doi: 10.1371/journal.pone.0242598 (PMC7703992; doi:10.1371/journal.pone.0242598)
Supplement: S3 Fig — Receiver operating characteristic (ROC) curve analysis was applied to determine area under curve (AUC) and positive cut-off for SGM infection prediction by detecting anti-GPL core IgG level. Red arrow represents cut-off point on ROC curve. (DOCX) [file pone.0242598.s003.docx]

**S3 Fig. Determination of positive cut-off to discriminate slow growing mycobacteria (SGM) from rapid growing mycobacteria (RGM) by quantification of** **anti-GPL core IgG.** Receiver operating characteristic (ROC) curve analysis was applied to determine area under curve (AUC) and positive cut-off for SGM infection prediction by detecting anti-GPL core IgG level. Red arrow represents cut-off point on ROC curve.
