# Supplementary material for: Evaluation of plasma anti-GPL-core IgA and IgG for diagnosis of disseminated non-tuberculous mycobacteria infection
Source: PLoS One. 2020 Nov 30;15(11):e0242598. doi: 10.1371/journal.pone.0242598 (PMC7703992; doi:10.1371/journal.pone.0242598)
Supplement: S1 Table — (DOCX) [file pone.0242598.s004.docx]

**S1 Table. Specificity, PPV and NPV of positive cut-off value to distinguish NTM infection patients from different group of subjects without NTM infection**

|  | **Positive cut-off value for NTM** | **Subjects with other infections**  **(MTB + BP, n = 37)** | |  | **Subjects with tuberculosis**  **(MTB, n = 18)** | |  | **Healthy subjects**  **(HC, n = 30)** | |
| --- | --- | --- | --- | --- | --- | --- | --- | --- | --- |
|  |  | **No. of negative samples / total no. of samples** | **% specificity (95%CI)** |  | **No. of negative samples / total**  **no. of samples** | **% specificity (95%CI)** |  | **No. of negative samples / total**  **no. of samples** | **% specificity (95%CI)** |
|  |  |  |  |  |  |  |  |  |  |
| **Anti-GPL core IgA** | > 0.352 | 24/37 | 72.97  (55.88 – 86.2) |  | 12/18 | 66.67  (40.99 – 86.66) |  | 18/30 | 66.67  (47.19 – 82.71) |
| **Anti-GPL core IgG** | > 4.140 | 32/37 | 86.49  (71.23 – 95.46) |  | 16/18 | 88.89  (65.29 – 98.62) |  | 28/30 | 93.33  (77.93 – 99.18) |
|  |  | **No. of NTM samples / total no. of positive samples** | **% PPV (95%CI)** |  | **No. Of NTM samples / total**  **no. of positive samples** | **% PPV (95%CI)** |  | **No. of NTM samples / total**  **no. of positive samples** | **% PPV (95%CI)** |
|  |  |  |  |  |  |  |  |  |  |
| **Anti-GPL core IgA** | > 0.352 | 32/42 | 76.19  (60.55 – 87.95) |  | 32/38 | 84.21  (68.75 – 93.98) |  | 32/42 | 76.19  (60.55 – 87.95) |
| **Anti-GPL core IgG** | > 4.140 | 32/37 | 86.49  (71.23 – 95.46) |  | 32/34 | 94.12  (80.32 – 99.28) |  | 32/34 | 94.12  (80.32 – 99.28) |
|  |  | **No. of non-NTM samples / total no. of negative samples** | **% NPV (95%CI)** |  | **No. of non-NTM samples / total**  **no. of negative samples** | **% NPV (95%CI)** |  | **No. of non-NTM samples / total**  **no. of negative samples** | **% NPV (95%CI)** |
|  |  |  |  |  |  |  |  |  |  |
| **Anti-GPL core IgA** | > 0.352 | 27/30 | 90.00  (73.47 – 97.89) |  | 12/15 | 80.00  (51.91 – 95.67) |  | 20/23 | 86.96  (66.41 – 97.22) |
| **Anti-GPL core IgG** | > 4.140 | 32/35 | 91.43  (76.94 – 98.20) |  | 16/19 | 84.21  (60.42 – 96.62) |  | 38/31 | 90.32  (74.25 – 97.96) |

NTM; non-tuberculous mycobacteria, MTB; *M. tuberculosis*, BP; *B. pseudomallei*, HC; healthy control.

PPV; positive predictive value, NPV; negative predictive value, CI; confidence interval.
