# Supplementary material for: Evaluation of plasma anti-GPL-core IgA and IgG for diagnosis of disseminated non-tuberculous mycobacteria infection
Source: PLoS One. 2020 Nov 30;15(11):e0242598. doi: 10.1371/journal.pone.0242598 (PMC7703992; doi:10.1371/journal.pone.0242598)
Supplement: Attachment — Submitted filename: CommentsGLP.pdf [file pone.0242598.s005.pdf]

The aim of the manuscript was to evaluate surplus plasma samples from Thai patients suspected of disseminated NTM with an anti-GPL-core IgA kit and the modified kit to also detect IgG to compare the diagnostic potential of both isotypes.

The authors' approach is interesting, however serology IgA-GPL has some limitation, that is import for clinicians. As described by the authors and others GLP is present in MAC, RGM, including M. abscessus, and IgA-GPL core ELISA cross-reactivity between MAC and RGM in pulmonary cases is reported. It is well known that the drug treatment regime is different for MAC and M. abscessus. However, both IgA and IgG tests failed to distinguish them in disseminated NTM cases. Even significantly higher IgG median reactivity was described for RGM the test weakness remains because the number of tested samples is too small.

According to the reference cited by the authors, a meta-analysis study in the diagnostic accuracy of IgA-GPL core in MAC pulmonary diseases was 0.696 (95% CI 0.621–0.761) and 0.906 (95% CI 0.836–0.951), for sensitivity and specificity, respectively. Looking at the results of the present study the IgA sensitivity (61.7) and specificity (95) are similar if use a higher cut off for disseminated NTM cases. This shows the IgA-GPL core accuracy is consistent in pulmonary MAC and disseminated NTM diseases. This discussion is missed.

On the other hand, the IgG test showed higher reactivity distribution in disseminated NTM samples, but the other infections disease and control also showed dispersion and cross-recativity. As the number of MTB and BP samples is too small, thus may cause bias in the test specificity. How do the authors explain this cross-reactivity?

All this makes the authors' assumption “Taken together, the application of anti-GPL-core antibody detection provides a novel additional option for clinicians to diagnose and manage disseminated NTM infected patients” speculative. The authors should discuss what can be the role of the biomarkers-based assays for the diagnosis of disseminated NTM and treatment management considering the limitations of the test.

The major limitation of the study is the sample size of MTB and BP cases. With few subjects included is difficult to really assess the role of the IgG test, although for IgA the accuracy was consistent for both MAC pulmonary disease and disseminated NTM.

Other comments:

Line 104-104 - Consider to change: To detect anti-GPL-core IgG, the antigen adsorbed plate of the IgA kit was used to perform the modified test. Briefly, ...

Line 107 - Biotinylated ....anti-human IgG (BD Biosciences, USA). It was made in what animal?

Line 112-113 - ... Results were analyzed with in-house standard curve and reported as arbitrary unit/ml (AU/ml). How did you that? Did you use the software? Manual mathematic calculation? Which plasma reference was used?

Line 128 – 129 - --- biotinylated anti-human IFN-γ (BD Biosciences, USA). The conjugate was titrated?

Seems that it was used surplus plasma samples of suspected disseminated NTM cases from a Hospital biorepository and the control was freshly collected. However, the authors did not comment on the storage condition of the samples. This is an important issue in serology because Ab degradation can alter the ELISA results.

Line 159 - .....plasma samples from routine assay were collected from storage at..... Inform the storage condiction (temperature? suffered freeze-thaw?)
